# Disease-Modifying Treatment Options in Very Early Onset Multiple Sclerosis—What Choices Are There for Onset Under 5 Years of Age? A Systematic Review

**DOI:** 10.3390/jcm14228133

**Published:** 2025-11-17

**Authors:** Dana Craiu, Alice Denisa Dica, Cristina Pomeran, George Pescaru, Shay Menascu, Mihaela Simu

**Affiliations:** 1 Pediatric Neurology Discipline, Department of Neurosciences, Carol Davila University of Medicine and Pharmacy, 050474 Bucharest, Romania; denisa_dica@yahoo.com (A.D.D.); or cristina.pomeran@umfcd.ro (C.P.); 2 Pediatric Neurology Center of Expertise of Rare Pediatric Neurological Disorders, Alexandru Obregia Hospital, 041914 Bucharest, Romania; 3 Romanian Group of Undiagnosed Rare Disorders of the Institute of Research and Development in Genomics, 020021 Bucharest, Romania; 4 Neuroimmunology and Demyelinating Pediatric Service, Multiple Sclerosis Center, Sheba Medical Ceter, Tel Aviv 6997801, Israel; shay.menascu@sheba.health.gov.il; 5 Sackler Faculty of Medicine, Tel Aviv University, Tel Aviv 6997801, Israel; 6 Department of Neuroscience, University of Medicine and Pharmacy “Victor Babes”, 300041 Timisoara, Romania; mihaelasimu6713@gmail.com; 7 Second Neurology Clinic of “Pius Brînzeu” Emergency Clinical County, Hospital, 300723 Timisoara, Romania

**Keywords:** very early onset multiple sclerosis, disease-modifying treatment, pediatric-onset multiple sclerosis (POMS), Natalizumab, interferon, immunoglobulins, acute treatment

## Abstract

**Background/Objectives:** Very early pediatric-onset multiple sclerosis (POMS) is rare; clinical studies using disease-modifying treatments (DMTs) have not been performed. Clinicians rely on studies performed at older ages. This review resulted from difficulties faced by clinicians and the off-label use of DMTs at this age. **Methods**: A literature review of studies dated between 1982 and 2025 on very early POMS, specifically with onset before age 5, has been performed, searching for outcomes without or with DMTs. The curated database of the selected patients was analyzed using computed descriptive and integrated cohort-level estimates. The clinical, paraclinical, treatment, and outcome characteristics were analyzed. Statistical analysis used JASP, with GenAI-assisted verification. The treatment outcome of a 16-year-old patient with very early POMS starting at 2 years 4 months that consecutively received interferon, immunoglobulin, and Natalizumab is presented. **Results**: A total of 101 patients with very early POMS presented, at onset, with ataxic syndrome (57.4%), pyramidal syndrome (41.4%), ophthalmoplegia (10.3%), and optic neuritis (6.9%). In evolution, 22.7% had seizures. Half of the patients were not treated. Among those treated, acute steroid therapy was administered; 11 received the DMTs interferon, Glatiramer acetate, Dimethyl fumarate, and Azathioprine (three), with only two high-efficacy therapies (Natalizumab and Rituximab). Our patient had partial remission under interferon, relapses when stopped and replaced by immunoglobulin and 9 years relapse-free interval when Natalizumab was introduced. **Conclusions**: Early treatment with high-efficiency DMTs should be considered in very early POMS; association with known increased neuroplasticity at this age may improve prognosis, allowing good recovery of acquired disability.

## 1. Introduction

The prior literature typically defines POMS as occurring in patients < 18 years and considers those <10 years as early-onset cases. In this work, we define very early POMS cases with onset before 5 years of age. POMS represents 3% to 5% of all multiple sclerosis cases, with less than 1% displaying overt symptoms before the age of 10 years (early POMS), comprising 0.09/100,000 of the population [1]. Prevalence and incidence of very early POMS are unknown [2,3,4]. Due to the very low incidence of MS at this age, there are no powerful studies concerning DMT benefits or adverse effects. Due to its rarity, only isolated case reports or small case series exist [5,6]. Therefore, clinicians treating these cases need information to manage diagnosis and treatment for very early POMS. DMTs are off-label and not covered by insurance policies in many countries; the patients receive only acute therapy and sometimes intravenous immunoglobulins, with uncertain efficacy and accumulation of disability [6].

Authors of this review faced difficult decisions for treating a patient with very early POMS in a female with onset at age 2 years 4 months. She initially had frequent episodes of neurological involvement treated with acute steroid therapy, with complete remission. DMTs used in sequence (Interferon, intravenous immunoglobulins (IVIG), and Natalizumab) have different effects. The case presentation is available in the Section 3.3 clinical vignette.

This case is the reason for the extensive literature review presented in this publication, aiming to offer our pediatric colleagues a summary of the published cases of very early POMS, including clinical aspects and treatment choices.

## 2. Materials and Methods

### 2.1. Literature Search

**Figure 1:**
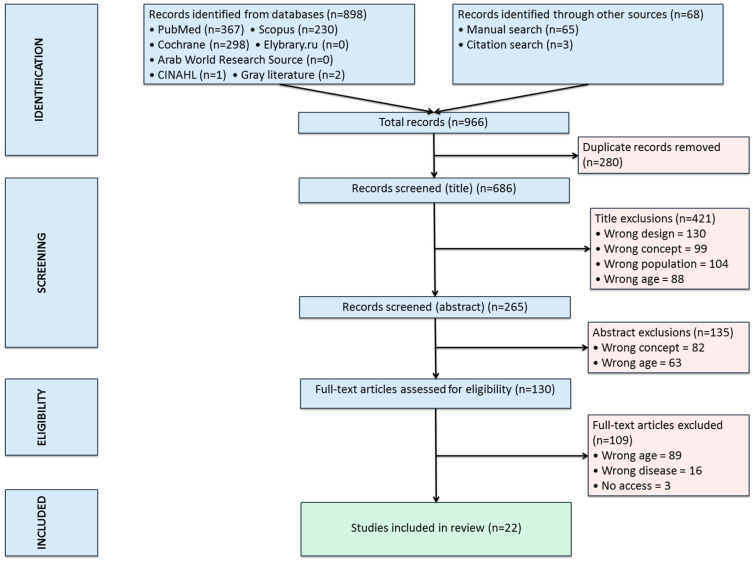
PRISMA flow chart.

A systematic review was conducted in accordance with the Preferred Reporting Items for Systematic Reviews and Meta-Analyses (PRISMA) guidelines, in accordance with the Preferred Reporting Items of Systematic Review (PRISMA) guidelines [7]. The completed PRISMA checklist is provided in Table S1. The checklist identifies the section in the manuscript where each item is reported. The selection process is depicted in the Flow Diagram in Figure 1.

PubMED, Scopus, Cochraine, Elybrary.ru, Arab World Research Source, CINAHL, and OpenGrey were searched using following search terms: ((“Multiple Sclerosis” [Mesh] OR “multiple sclerosis” [tiab] OR “pediatric multiple sclerosis” [tiab]) AND (“Child, Preschool” [Mesh] OR “Infant” [Mesh] OR child [tiab] OR children [tiab] OR toddler [tiab] OR preschool [tiab] OR “younger than 5” [tiab] OR “under 5” [tiab] OR “before age 5” [tiab]) AND (“Therapeutics” [Mesh] OR “Drug Therapy” [Mesh] OR “Treatment Outcome” [Mesh] OR treatment [tiab] OR therapy [tiab] OR corticosteroids [tiab] OR interferon [tiab] OR “disease modifying therapy” [tiab] OR DMT [tiab] OR Glatiramer [tiab] OR Rituximab [tiab] OR cyclophosphamide [tiab] OR “Dimethyl fumarate” [tiab]) OR (“Natalizumab” [Mesh] OR Natalizumab [tiab])).

Articles found after a manual search in the hospital literature database (selected articles on the topic) and in the references of the selected articles were added.

Article selection was performed independently by two reviewers (P.G. and D.A.). Inclusion and exclusion criteria were applied consistently at the title, abstract, and full text to ensure transparency and reproducibility.

The inclusion criteria were as follows: (1) age at onset below 5 years; (2) publication date after 1982, in order to include the use of paraclinical investigations introduced by the Poser criteria (with subsequent selection of the cases using IPMSSG and McDonald 2010 criteria) [8,9,10]; (3). Design of the study: case reports, series of cases, clinical trials, and literature review articles; (4) Publication languages: English, Spanish, Arabic, Russian, and Chinese. Google Translate was used to translate from any language to English.

Studies that did not fulfill the contemporary diagnostic requirements for multiple sclerosis at the time of publication, studies that failed to provide clinical and paraclinical details of individual patients or cohorts over 5 years of age were excluded.

Generative artificial intelligence (GenAI)-ChatGPT version 5.0- has been used to generate the PRISMA flow diagram, Figure 1 (to illustrate the process of study identification, screening, eligibility assessment, and final inclusion).

A relevant case study of multiple sclerosis with very early onset was described as an example of different DMTs used off-label for age; evolution is described and compared with literature findings.

### 2.2. Data Analysis

The authors analyzed the following information: (1) demographic and clinical data, (2) paraclinical data, (3) treatment at episode as well as DMTs and their effect. For more information on treatment and DMTs, see Table S2, containing collected data classification; patients and their characteristics are presented in Table S3, which delineates patient characteristics from selected articles.

Data extraction was performed independently by two reviewers (G.P. and A.D.D.) using a standardized data extraction form developed in Microsoft Excel. Extracted variables included demographic information, clinical features, paraclinical investigations, treatments (acute and disease-modifying therapies), and outcomes (Table S2). Any discrepancies between reviewers were resolved through discussion and, if necessary, consultation with a third reviewer (D.C.). No automation tools were used for data extraction.

For each outcome, studies were grouped narratively according to clinical, paraclinical, and therapeutic domains. Only studies that reported data relevant to the predefined outcomes were included in each synthesis. When data were incomplete or presented in incompatible formats, they were reported descriptively without transformation. Results were summarized using tables.

### 2.3. Statistical Analysis

The statistical analyses and figures were produced in JASP version 095.1. Verification, cross-checks, and iterative re-analysis of the results were assisted by GenAI under author verification; GenAI was only used to support calculations, code expressions, and interpretation, and not to generate or alter primary data.

Descriptive results (counts and percentages) were calculated for demographic, clinical, paraclinical, and treatment data.

The authors hypothesized a strong association between younger onset age and a higher incidence of ataxia or seizures at onset. Therefore, Pearson and Spearman rank correlations with 95% CIs were run after excluding records with unknown evolution and cohorts without individual-level data. For ataxia, age association was adjusted for cerebellar lesions and seizures; age association was also adjusted for cortical/subcortical lesions using binomial logistic regression analysis. Models were restricted to cases with magnetic resonance imaging (MRI) data.

The hypothesis whether incomplete remission differed by treatment exposure was examined with χ^2^ test across three groups (no treatment, steroid therapy only, and steroid therapy + DMT) for complete or incomplete remission, followed by binomial logistic regression with incomplete remission as outcome (odds ratio with 95% CIs; *p* value < 0.05).

## 3. Results

### 3.1. Literature Search

A total of 966 records were identified. Duplicates and articles not fulfilling inclusion criteria were excluded; 22 studies remained for analysis [5,11,12,13,14,15,16,17,18,19,20,21,22,23,24,25,26,27,28,29,30,31] (Figure 1 and Table S3).

### 3.2. Data Analysis

#### 3.2.1. Demographic and Clinical Data

A total of 101 patients were identified (Table 1). The female-to-male ratio was 1.4:1. The mean age at onset was 36 months (range: 10 months–5 years). The mean number of relapses was 4.1, with an average of 2.3 relapses within the first two years of life.

Onset signs. The most frequent presenting syndromes were ataxic syndrome (57.4%), pyramidal syndrome (41.4%), fever with or without altered consciousness (17.2%), ophthalmoplegia (10.3%), and optic neuritis (6.9%).

Signs in evolution. Ataxic gait was the most common symptom (42.9%), followed by hemiparesis (35%) and seizures (22.7%) (Table 2).

In the majority of cases, both the initial presentation and early relapses were monosymptomatic (74.3%).

An onset resembling ADEM, with fever, lethargy, impaired consciousness, and vomiting, was reported in 17.8% of cases, all of whom developed classical recurrent remitting multiple sclerosis (RRMS) later on.

#### 3.2.2. Paraclinical Data

- Imaging: MRI was performed in 98 cases. Supratentorial lesions predominated (57.4%), most commonly periventricular (59.1%); infratentorial lesions were primarily located in the brainstem (32.7% of all cases) (Table 3). Cerebellar lesions were observed in 10 cases (10.2% of all cases) and spinal lesions in 12 cases (10.2% of all cases). Six patients underwent computer tomography (CT) examination (three had only a CT-scan), of which five revealed hypointense lesions. Two of the patients had a normal CT but abnormal IRM at the follow-up. The other one with normal CT had MS characteristic lesions that were confirmed by autopsy.

#### 3.2.3. Cereb

- Cerebrospinal fluid (CSF) analysis performed in 99 cases showed pleocytosis (45 out of 999 cases/45.5%), hyper-proteinorrachia (27 out of 99 cases/27.3%), positive oligoclonal bands (32 out of 99 cases/32.3%), elevated IgG index (5 of 9 tested/55.5%) and anti-myelin basic protein antibodies (3 out of 3 cases/100%).
- Visual evoked potentials (VEPs) were abnormal in 10 out of 30 patients (33,3%). Autopsies were performed on two deceased patients, revealing multiple small sclerotic lesions, some with cystic components, distributed within the white matter, predominantly supratentorial periventricular.

#### 3.2.4. Treatment

- Regarding treatment, 43 of all treated patients (95.6%) received steroids for at least one relapse (Table 4). In most cases, high-dose intravenous methylprednisolone was used, with or without subsequent tapering with oral prednisone.
- Three additional patients received intravenous immunoglobulin (IVIG) and steroids.
- Disease-modifying therapies were initiated in 11 cases (24.4% of all treated patients), of which 6 received low-efficacy agents (4 interferon, 1 Dimethyl fumarate, and 1 Glatiramer acetate) and 2 received high-efficacy agents (1 Natalizumab and 1 Rituximab). Azathioprine was administered in three cases.

#### 3.2.5. Outcomes

- Complete remission was documented in 88% of cases.
- Poor outcomes were observed in 12 cases, characterized by multiple relapses with incomplete recovery, progressive course, or lack of remission. Two deaths occurred, both in patients from the pre-2001 cohort who had not received treatment. Among patients with incomplete remission, 8 were treated with steroids, and 1 with steroids and Dimethyl fumarate.
- Escalation to higher efficacy therapies (Rituximab, Natalizumab, and Azathioprine) occurred in three cases, with adequate disease control achieved in the first two cases.

#### 3.2.6. Statistical Analysis of Treatment Outcomes

No significant associations were detected between age at onset and symptoms at onset: ataxia (Pearson r = 0.364, *p* = 0.088, 95% CI −0.057 to 0.675) or seizures (Pearson r = −0.261, *p* = 0.229, 95% CI −0.608 to 0.169). MRI lesions may introduce a bias for epilepsy (the cortical lesions) and ataxia (the cerebellar and spinal lesions). In the adjusted logistic models (with MRI lesions as a confounder), the statistical associations did not change.

**Figure 2:**
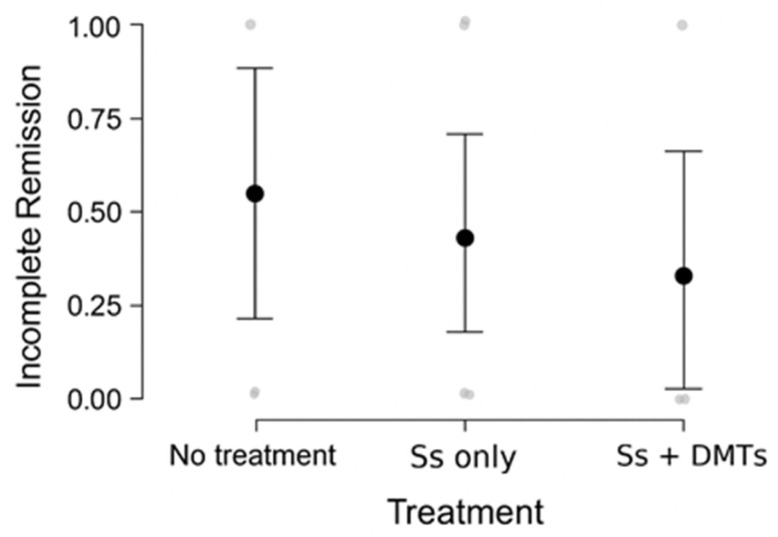
Predicted probability (95% CI) of incomplete remission by treatment group from binomial logistic regression. The plot illustrates a decreasing probability for incomplete remission with increasing treatment intensity. Big black dots represent model-based estimates; bars indicate 95% CIs; light dots show individual remission outcomes tendencies (jitter). Ss = steroids.

The contingency test assessing remission in treated patients was not statistically significant (χ^2^ = 1.26, *p* = 0.53), although the proportion for incomplete remission has a decreasing trend in relation to more complex treatments: 50% incomplete remission in the untreated group, 31% for patients treated with steroids only, and 17% for steroids and DMTs. Consistently, logistic regression using the steroids and DMTs group as the reference estimated higher odds (but non-significant) for incomplete remission for the steroids-only group (OR = 2.22, *p* > 0.05) and the no-treatment group (OR = 5.00, *p* > 0.05). Overall, results do not show statistically significant associations, but the statistical model estimates that adding a DMT on top of corticosteroids likely reduces the chance of incomplete remission, consistent with a clinically favorable effect (Figure 2).

### 3.3. Clinical Vignette

**Figure 3:**
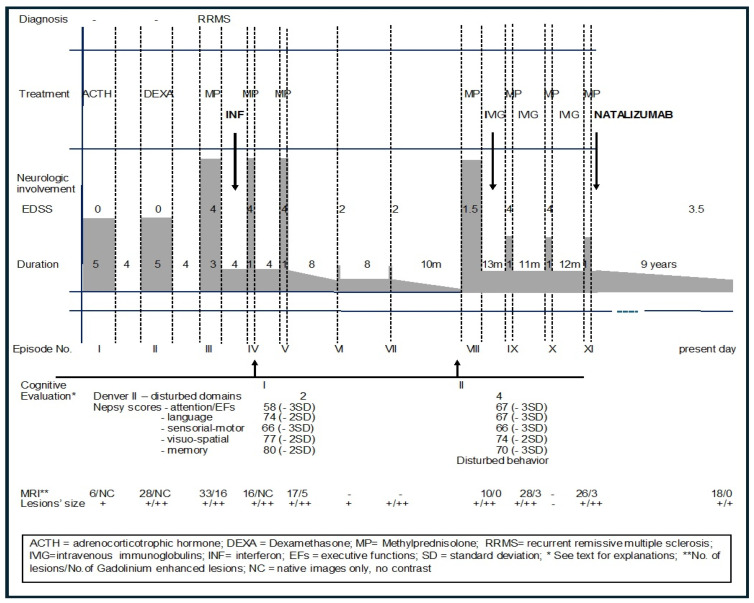
Patient clinical and imaging evolution correlated with treatment. Vertical dashed lines = time between episodes; downward arrows = treatment initiation; upward arrows = cognitive evaluation; grey area = neurological involvement during episodes and after episode remission.

A 16-year-old female patient is presented. She was born by vaginal delivery after an uncomplicated pregnancy and had normal milestone achievement. The family history was unremarkable for the patient’s disease. She had 11 episodes of neurological involvement with a stormy onset at 2 years 4 months. As a characteristic, her episodes were long at onset, with complete recovery over a short duration (but more than 30 days). After the third episode, residual neurological symptoms were noted. The clinical symptoms at onset and subsequent episodes are listed in Table 5 and Figure 3.

#### 3.3.1. Workup

The initial symptoms were rapidly progressive gait and equilibrium disturbances, and irritability without a prior history of infectious disease. In another hospital, a suspicion of ADEM was raised, but clinical and paraclinical data, including imaging and clinical evolution, with new relapses, led to the diagnosis of RRMS after the third episode. Table 6 shows the workup and differential diagnosis.

Infectious and inflammatory immune disorders were ruled out.

**Figure 4:**
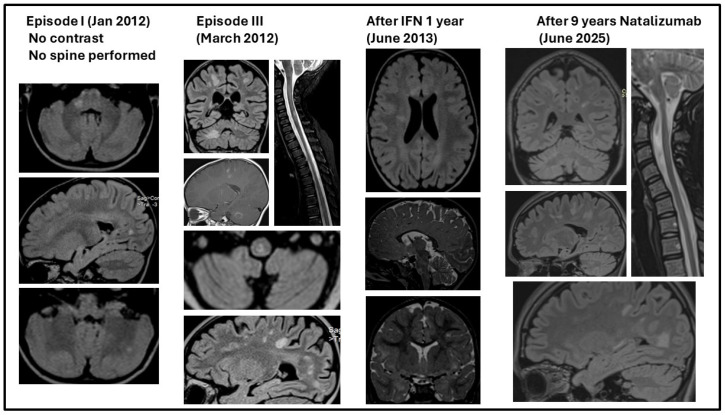
MRI at onset and in evolution.

The diagnosis of RRMS was established at that time based on Mc Donalds criteria 2010 [9]: she had more than two attacks proving dissemination in time (DIT) and objective clinical and MRI evidence of dissemination in space (DIS). On MRI, she had lesions in all four regions—periventricular, juxtacortical, infratentorial, and spinal cord regions—proving DIS; these lesions had different ages, proving DIT (Figure 4).

#### 3.3.2. Evolution Under Treatment

The patient received steroid therapy at every acute event.

Episodes I and II: Small doses of ACTH (0.33 mg/day for 14 days), followed by Prednisone (0.5 mg/kg), for 7 days, were administered in the county hospital. She had a complete remission 5 weeks after onset.

The second episode started after 30 days (Table 5, Figure 3). No other investigations were carried out by the local physician. Treatment with Dexamethasone 8 mg/day for 3 days and Synachten 0.5 mg/day for 7 days was followed by complete remission within 5 weeks and another 35 days without neurological symptoms. MRI was performed at onset and after remission (Figure 4) of the third episode, showing an increased number of MRI lesions.

For the third episode, she was admitted to our clinic with cerebellar syndrome (severe ataxia and saccadic speech), pyramidal syndrome (left more than right), and irritability. Treatment included acute pulse therapy with Methylprednisolone 30 mg/kg/day for 6 days: the patient was started on DMT (disease-modifying treatment) off-label—Interferone Beta-1a 22 µg/dose, 3 doses/week—premedication with paracetamol was initiated, and no adverse reactions were recorded. Periodical follow-up (clinical and biological) showed IFN was well tolerated. She had another two mild and short relapses (1 week each) with 4 weeks intervals between episodes, followed by another two relapses of 2 days each with 8 weeks intervals between them (Table 6, Figure 3). With the IFN treatment, the Expanded Disability Status Scale (EDSS) score decreased continuously from 4 to 2 and even to 1.5. The sponsor could not support the donation of IFN anymore due to legal constraints, and it was discontinued. She relapsed dramatically after 10 weeks (episode VIII), and she was initiated on IVIG (intravenous immunoglobulins) 2 g/kg/cure infused in 3 days, monthly. As a result, she had mild relapses at intervals of 11–13 weeks lasting no more than 1 week each, but EDSS remained 4 also during the “free” intervals between relapses.

Due to international collaboration with colleagues with experience in treating pediatric patients with Natalizumab, the patient was initiated at age 7 years on Natalizumab, 300 mg/dose, with monthly administrations. Check-up for JCV (John Cunningham virus, human polyomavirus 2) has been performed before and then monthly, and MRI was performed for PML (progressive multifocal leukoencephalopathy) at each relapse and yearly for follow-up; both JCV and MRI were performed during IFN and Natalizumab therapies. No relapses were recorded after Natalizumab initiation during the next 9 years, until present; the EDSS score decreased to 3.5 (persistent disability).

Cognitive involvement. Parents did not notice changes in the patient’s cognitive performance. At age 3 years, she was evaluated (Romanian validated tests). Denver II showed delayed development in personal–social and gross motor domains, with normal functioning for language and fine motor and adaptive behavior domains. The Nepsy test showed decreased scores in all domains: attention/executive functions 58, -3SD (standard deviation); language 74, -2SD; sensory-motor 66, -3SD; visual-spatial 77, -2SD; memory 80, -2SD. During testing, she collaborated with psychologists but showed severe inattention and a mild lack of interest.

She was reevaluated at age 4 years 1 month by the same psychologists. Denver II showed delayed development in all four domains. Nepsy proved decreased scores for language, visual–spatial, and memory compared with the previous examination: attention/executive functions 67, -3SD; language 67, -3SD; sensory–motor 66, -3SD; visual–spatial 74, -2SD; memory 70, -3SD (Figure 3). The patient showed opposing and provoking behavior, inappropriate verbal and emotional reactions, and difficulties in maintaining attention, resulting in a lack of collaboration during testing.

She was reevaluated at age 15.5 years, has an average normal IQ of 91, with social and adaptive impairment, and has been attending high school (admitted by contest). No complex evaluation was performed.

## 4. Discussion

Multiple sclerosis is extremely rare before the age of 5 years. Isolated case reports and small case series have been published, and some cases are documented in national registries [24,33].

The case reported in the vignette is an MS patient with an exceptionally early onset (age: 2 years 4 months). An even earlier subclinical onset with unnoticed clinical signs due to the small age of the patient is speculated. As described, an even earlier onset (10 months old) was previously reported by other groups [24]. It is unknown whether very early-onset multiple sclerosis shares the same pathophysiology as in adult patients or whether distinct underlying biological mechanisms may trigger it. It was hypothesized, but not yet proven, that copy number variation (CNV) may influence genes in large genomic regions involved in disease mechanisms and contribute to very early onset [34]. Genetic background may play a major role in early-onset MS, and further studies are expected.

Age-specific incidence: While pediatric-onset multiple sclerosis (POMS) accounts for 3–5% of all MS cases, with fewer than 1% developing the disorder under the age of 10, there is substantial variability in incidence rates based on geographic location and environmental exposures. Regions with higher latitude, such as Scandinavia and Canada, demonstrate significantly higher pediatric MS prevalence due to possible correlations with lower vitamin D levels [2]. These data highlight the importance of understanding regional susceptibility alongside diagnostic patterns, which show that early-onset MS might still be underdiagnosed in populations from lower-income countries.

A marked discrepancy can be observed between the high number of pediatric MS cases with onset before 5 years of age reported between 1980 and 2000, compared with those, albeit fewer, published between 2001 and 2025. This phenomenon can largely be attributed to the absence of modern diagnostic criteria before the year 2000, limited availability of advanced neuroimaging, and, most importantly, the still not yet described related neuroinflammatory demyelinating disorders such as neuromyelitis optica spectrum disorder (NMOSD), MOG antibody-associated disease (MOGAD), or autoimmune encephalitis [35,36].

In the present review, we identified 19 published patients who had been diagnosed with MS but who no longer meet contemporary diagnostic criteria [37,38,39,40,41,42,43,44,45,46]. All of these cases were reported between 1981 and 1994. Among them, three patients would now probably be classified as having MOGAD, two as having ADEM, one as having NMOSD, and one as having autoimmune encephalitis, due to clinical and MRI aspects. For the majority, however, insufficient clinical and paraclinical data were available to fulfill current diagnostic requirements for MS, although the diagnosis cannot be definitively excluded. These articles were excluded from our review.

In accordance with the diagnostic criteria for multiple sclerosis, it is essential to exclude other possible pathologies that could explain the symptomatology, particularly MOGAD in the young pediatric population. MOGAD is an inflammatory demyelinating disorder of the central nervous system associated with autoantibodies against the myelin oligodendrocyte glycoprotein. Transverse myelitis, optic neuritis, ADEM, and cerebral cortical encephalitis are the most typical clinical presentations [47]. The disorder was first described in 2007 [48]. Currently, there are clear criteria to support the diagnosis, including the detection of MOG antibody [49]. However, the identification of MOG antibodies became available on a large scale after 2014 [50]. In our literature cohort, 75% of all cases diagnosed after 2014 were tested for MOG and AQ4 antibodies. Unfortunately, many patients had been published before MOGAD was well defined and could not be evaluated for this diagnosis, which represents a limitation of the present study. Nevertheless, all selected patients, not tested for MOG and AQ4 antibodies, fulfilled the IPMSSG diagnostic criteria, with at least two non-encephalopathic episodes at intervals of more than 30 days affecting at least two areas of the CNS, or at least one non-encephalopathic clinical event at least 3 months after an ADEM-like onset with new MRI lesions suggestive of multiple sclerosis [10].

Our case presented the first demyelinating episode in 2012, when the detection of MOG antibodies was not available in our country. However, she exhibited a typical MS evolution, with multiple non-ADEM relapses, new circumscribed lesions suggestive of multiple sclerosis, and the diagnosis was confirmed by the IPMSSG [10]. She did not present bilateral optic demyelination or other clinical features that could have been considered red flags for MOGAD. Moreover, she showed a poor response to steroids and immunoglobulin (MOGAD cases frequently respond favorably).

Symptoms and Early MRI Trends: Ataxia was observed as the most frequent presenting manifestation in our cohort, consistent with prior studies indicating that cerebellar and brainstem involvement is more pronounced in younger MS patients [24,51,52]. Additionally, patients with seizure onset, although infrequent, displayed a suggestive association with cortical lesions. This aligns with previous reports linking epilepsy to MS regardless of age [53].

Treatment remains a challenge under 5 years of age due to the absence of clinical studies with DMTs. In the analyzed group, half of the patients were not treated. Among those treated, acute-phase steroid therapy was administered; DMTs were administered in 11 patients: most received low-efficacy agents such as interferon, Glatiramer acetate, or Dimethyl fumarate, three were treated with Azathioprine [15,24,25], and only two patients received high-efficacy therapies (Natalizumab or Rituximab) [5,18,28,31]. A patient reported by Sotgiu et al. with disease onset at age 5 years and treated with Natalizumab since age 5 years and 5 months had nearly complete remission with significant EDSS score improvement [31]. An Italian observational study using Natalizumab in MS pediatric patients showed good tolerability and good response when administered as early as 4 years of age [54,55].

A groundbreaking advancement in treating POMS with high-efficacy therapies is highlighted by the study of Menascu et al. [56]. The multicenter research focuses on outcomes in pediatric MS treated with Natalizumab, revealing significant benefits in reducing relapse rates and stabilizing disease over long-term follow-up. The study demonstrated a significant decrease in EDSS scores and relapse rates in children as young as 5 years of age during Natalizumab therapy. Reported adverse events were mild or moderate, and periodic John Cunningham virus (JCV) screening was implemented to mitigate the risk of progressive multifocal leukoencephalopathy (PML). These findings suggest that Natalizumab, though off-label for children under 18 years [57], may be a critical tool in cases of highly active POMS, particularly under rigorous monitoring protocols.

After a partial response when treated with interferon and no response with IVIG, our vignette patient similarly benefited from Natalizumab, initiated at 7 years of age, experiencing no relapses during a 9-year follow-up period. These results correlate strongly with Menascu et al.’s findings [56], supporting Natalizumab’s safety and efficacy for early-onset MS.

Early treatment with high-efficiency DMTs may improve prognosis in very early onset pediatric multiple sclerosis cases, especially because association with known increased neuroplasticity at this age, allowing good recovery of acquired disability [58].

Statistical analysis. Overall, our statistical modeling demonstrated trends favoring early introduction of high-efficacy disease-modifying therapies like Natalizumab in patients with very early onset multiple sclerosis. Moreover, interventions targeting children under 5 years may provide a critical neurological therapeutic window due to heightened neuroplasticity at these ages.

To deepen understanding, future studies should focus on global multicenter efforts to consolidate real-world pediatric data into standardized cohorts. This approach would better define the molecular mechanisms, genetic predispositions, and therapeutic responses specific to very early-onset multiple sclerosis. Integrating multicenter approaches, such as that described by Menascu et al. [56], could further optimize strategies for long-term disability prevention and improved quality of life in these pediatric populations.

## Figures and Tables

**Table 1:** Demographic features and follow-up.

Features	
**Sex**	
Male	42
Female	59
F:M ratio	1.47:1
**Age at onset (mo.) mean ± SD (range)**	36 ± 13.29 (10–60)
**Mean total number of attacks**	4.1
**Mean number of attacks in the first 2 years**	2.3
**Follow-up time (mo.) mean (range)**	26.4 (10–48)

**Table 2:** Clinical features and presentation during course of disease.

Features	Onset (% of Cases)	Evolution (% of Cases)
**Symptoms**		
Ataxia	57.4%	42.9.%
Pyramidal	41.4%	47.5%
Fever ± lethargy/altered consciousness	17.2%	4.9%
Ophthalmoplegia	10.3%	3.9%
Optic neuritis	6.9%	21.7%
Seizures	4.3%	22.7%
Neurogenic bladder	3.9%	3.9%
Other cranial nerve palsies	3.4%	19.8%

**Table 3:** Diagnostic work-up summary.

Features	% of All Cases	% of Tested Cases
**MRI**		
Supratentorial lesions	**57.4%**	**59.1%**
Periventricular	57.4%	59.1%
Cortical/subcortical	24.7%	25.5%
Infratentorial lesions	**36.6%**	**37.7%**
Brainstem	31.7%	32.7%
Spinal cord	11.9%	12.2%
Cerebellum	9.9%	10.2%
**CSF**		
Normal CSF	11.9%	12.1%
Oligoclonal bands (positive)	31.7%	32.3%
Pleocytosis	44.5%	45.5%
Elevated protein	26.7%	27.3%
**VEPs**		
Abnormal VEPs	9.9%	33.3%
Normal VEPs	19.8%	66.7%

**Table 4:** Treatment overview.

Features	Number	% of All Cases	% of Treated Cases
**Steroid therapy**	44	43.5%	80%
Methylprednisolone	26	25.7%	47.3%
Prednisone/prednisolone	17	16.8%	30.9%
Other steroids	3	2.9%	3.7%
**Intravenous immunoglobulin**	3	2.9%	3.7%
**Disease-modifying therapy**	11	10.9%	20.0%
Interferons	4	4.0%	7.3%
Azathioprine	3	2.9%	3.7%
Dimethyl fumarate	1	1.0%	1.8%
Glatiramer acetate	1	1.0%	1.8%
Natalizumab	1	1.0%	1.8%
Rituximab	1	1.0%	1.8%

**Table 5:** Detailed description of the episodes and treatment.

Episode	Age	Symptoms	Episode Duration	Type of Remission	Residual Symptoms	Free Interval After Episode	Treatment in Episode	DMT	Observations
I	2 y 4 m	Severe truncal ataxia Irritability	5 w	Complete	-	30 d	ACTH 0.5 mg/day—14 d Prednisone 0.5 mg/kg/day—7 d	-	
II	2 y 6 m	Severe ataxia (R > L CS) Irritability	5 w	Complete	-	30 d	Dexamethasone 8 mg/day—3 d ACTH 1/3 mg/day—14 d	-	
III	2 y 8 m	Severe ataxia (CS) L > R PS Irritability Saccadic speech (CS)	3 w	Incomplete	L pyramidal Mild ataxia	30 d	Methylprednisolone i.v. 30 mg/kg/day—6 d Tapered with Medrol IVIG 2 g/kg (in 6 d)	-	Diagnosis = RRMS
IV	2 y 10 m	Severe ataxia (CS) L > R PS Irritability	1 w	Incomplete	L pyramidal Mild ataxia	30 d	Methylprednisolone i.v. 30 mg/kg/day—5 d Tapered with Medrol	IFN beta-1a *	
V	2 y 11 m	Severe ataxia (CS) L > R PS Irritability	1 w	Incomplete	L pyramidal Mild ataxia	60 d	Methylprednisolone i.v. 30 mg/kg/day—5 d Tapered with Medrol		
VI	3 y 1 m	Irritability, mild ataxia	2 d	Incomplete	Mild L pyramidal	60 d	-		
VII	3 y 3 m	Irritability, mild ataxia	2 d	Incomplete	Mild L pyramidal	10 m	-		
VIII	4 y 1 m	Slight left intentional tremor (CS) Mild left hemiparesis (PS)	Not known	Incomplete	Mild L pyramidal	13 m	Methylprednisolone i.v. 30 mg/kg/day—5 d Tapered with Medrol	IVIG 2 g/kg/administration—monthly	
IX	5 y 2 m	Mild left hemiparesis	1 w	Incomplete	Mild L pyramidal	11 m	Methylprednisolone i.v. 30 mg/kg/day—5 d Tapered with Medrol		
X	6 y 1 m	Mild left hemiparesis	1 w	Incomplete	Mild L pyramidal	12 m	Methylprednisolone i.v. 30 mg/kg/day—5 d Tapered with Medrol		
XI	7 y 1 m	Mild left hemiparesis	1 w	Incomplete	Mild L pyramidal	9 y–present	Methylprednisolone i.v. 30 mg/kg/day—5 d Tapered with Medrol	Natalizumab 300 mg/dose every 28 d	No relapses since initiation

L = left; R = right; DMT = disease-modifying treatment; CS = cerebellar syndrome; PS = pyramidal syndrome; d = days; w = weeks; m = months; y—years; * Interferon beta-1a, 6.6 µg/dose, every other day, 2 weeks (7 doses), then 8.8 µg/dose, every other day, continuously, until now.

**Table 6:** Workup and differential diagnosis [32].

Differential Diagnosis	Observations	Investigation
NMO, recurrent	- No optic neuritis -Spinal cord involvement less than 3 spinal segments	AQP4 negative Oligoclonal bands negative
ADEM, recurrent	-No encephalopathy (except behavioral symptoms at first episode) Remission of initial symptoms followed by new symptoms after interval of 1 month (MS more probable), Miller et al. [29]	MRI—DIT, DIS, lesions typical for MS
Infectious diseases, including Borreliosis, HIV, HBV, EBV, cysticercosis	No fever No other organ involvement	LP—CSF and blood serology negative
Autoimmune disorders	No other organ involvement	Blood serology negative (ANA, anti-dsDNA antibodies, anti-Sm antibodies, serum complement)
Mitochondrial disorder	- No other organ involvement - Clinical evolution typical for MS - Treatment—efficacious	Lactic acid negative—blood and CSF; MRI—lesions typical for MS

NMO = neuromyelitis optica; AQP4 = serum aquaporin-4 autoantibodies; ADEM = Acute disseminated encephalomyelitis; MS = multiple sclerosis; MRI = magnetic resonance imaging; DIT = dissemination in time; DIS = dissemination in space; HIV = human immunodeficiency virus; HBV = Hepatitis B virus; EBV = Epstein–Barr virus; LP = lumbar puncture; CSF = cerebrospinal fluid; ANA = Anti-nuclear antibodies; anti-dsDNA = anti-double-stranded DNA antibody; anti-Sm = Anti-Smith antibody.

## Data Availability

Analyzed data are available online—Tables S2 and S3 (see above).

## Supplementary Materials

The following supporting information can be downloaded at: https://www.mdpi.com/article/10.3390/jcm14228133/s1. Table S1: PRISMA checklist; Table S2: Collected data classification; Table S3: Patients characteristics from selected articles.

## Abbreviations

The following abbreviations are used in this manuscript:

ADEM	Acute disseminated encephalomyelitis
ANAs	Anti-nuclear antibodies
Anti-dsDNA	Anti-double-stranded DNA antibody
anti-Sm	Anti-Smith antibody
AQP4	Serum aquaporin-4 autoantibodies
CI	Confidence interval
CSF	Cerebrospinal fluid
CT	Computer tomography
DIS	Dissemination in time
DIT	Dissemination in space
DMT	Disease-modifying therapy
EBV	Epstein–Barr virus
EDSS	Expanded Disability Status Scale
GenAI	Generative artificial intelligence
HBV	Hepatitis B virus
HIV	Human immunodeficiency virus
IVIG	Intravenous immunoglobulins
JCV	John Cunningham virus
LP	Lumbar puncture
MOG	Myelin oligodendrocyte glycoprotein
MOGAD	Myelin oligodendrocyte glycoprotein-associated disease
MRI	Magnetic resonance imaging
MS	Multiple sclerosis
NMO	Neuromyelitis optica
NMOSD	Neuromyelitis optic spectrum disorder
OCBs	Oligoclonal bands
POMS	Pediatric-onset multiple sclerosis
PRISMA	Preferred Reporting Items for Systematic Reviews and Meta-Analyses
RRMS	Relapsing remitting multiple sclerosis
Ss	Steroids
SD	Standard deviation
VEPs	Visual evoked potentials
