# Supplementary material for: Disease-Modifying Treatment Options in Very Early Onset Multiple Sclerosis—What Choices Are There for Onset Under 5 Years of Age? A Systematic Review
Source: J Clin Med. 2025 Nov 17;14(22):8133. doi: 10.3390/jcm14228133 (PMC12653428; doi:10.3390/jcm14228133)
Supplement: Supplementary file 1 [file jcm-14-08133-s001.zip › S1. Collected data classification.pdf]

Collected data classification

**Demographic data**	Sex<br>Age<br>Onset Age
**Clinical data**	Clinical signs at onset and in evolution<br>Number of episodes<br>Free symptoms interval<br>Follow-up time<br>Evolution
**Paraclinical data**	Neuroimaging (CT, IRM)<br>CSF parameters (Cell count, protein count, oligoclonal bands level, Ig G index, MAP antibody, AQ4 antibody)<br>Blood parameters (MOG antibody)<br>Visual evoked potentials<br>Morphopathology (if available)
**Treatment data**	Steroid therapy<br>Intravenous immunoglobulin<br>Disease modifying therapy (interferon, dimethyl fumarate, glatiramer acetate, natalizumab, rituximab, azathioprine)<br>Treatment response
