# Supplementary material for: Disease-Modifying Treatment Options in Very Early Onset Multiple Sclerosis—What Choices Are There for Onset Under 5 Years of Age? A Systematic Review
Source: J Clin Med. 2025 Nov 17;14(22):8133. doi: 10.3390/jcm14228133 (PMC12653428; doi:10.3390/jcm14228133)
Supplement: Supplementary file 1 [file jcm-14-08133-s001.zip › S2. Patients Characteristics.pdf]

Patient no.	Sex	Onset age	Symptoms	Workup	Tratament	Evolution	Refs.
1	F	5 y	First attack- ataxia and paraparesis<br>Second attack (8 m)- unilateral optic nevritis<br>Third attack (14 m)-cerebelar signs	MRI-lession in the white matter, thalamus, cerebel, medulla<br>CSF-oligoclonal bands, pleocytosis	Corticoterapy	Complete Remision	(Pérez-Fernández V. et al. 1999) [21]
2	M	3y6m	First attack- ataxia, impared consciousness<br>Frequent attacks 2-3/year with motor symptoms and persistent diplopia	MRI-periventricular and juxtacortical lesions with evolution in space and time<br>CSF-oligoclonal bands; MOG and AQP4 negative	Metilprednisolone pulsterapy<br>Dimethyl fumarate	Complete Remission	(Saijo N. et al.2022) [26]
3	F	5y	One attack-right hemiplegia, dysdiadochokinesia, sensitive ataxia	MRI-big round lesions periventricular, corpus callosum, leftr middle cerebellar peduncle and dorsal spinal cord – long lession	Metilprednisolone pulsterapy<br>Azathyoprine	Complete Remission	(Gargouri L. et al.2013) [15]
4	M	4y11 m	First attack-right hemiparesis<br>Second attack (10m)-Right hemiparesis+central right facial palsy	MRI-periventricular and medular short lesions with evolution in space and time<br>CSF-oligoclonal bands, MOG, AQP4 negative	Metilprednisolone pulsterapy<br>Rituximab	InComplete Remission<br>Complete Remission	(Arkar U. et. Al. 2024) [5]
5	F	3y6m	First attack-ataxia, paresthesia, vertigo<br>Second attack (2y)-vertigo,tinnitus<br>Third attack (1y)-weakness	MRI-periventricular and brainstem lesions<br>CSF-pleiocytosis, oligoclonal bands<br>Disturbet visual evoked	Metilprednisolone pulsterapy	InComplete Remission EDSS=5 (vertigo, poor vision)	(Ruggieri M. et al. 1999) [24]

			Fourth attack (6m)-paraplegia, GTCS<br>Fifth attack (10 m)-gait disturbances<br>Sixth attack (3y)-vertigo, optic neuritis	potentials			
6	F	3y8m	First attack-optic neuritis, diplopia<br>Second attack (1y)-ataxia, optic neuritis<br>Third attack (1y)-ataxia, optic neuritis, GTCS<br>Fourth attack (6m)-paraplegia, hypotonia	MRI-periventricular lesions<br>CSF-oligoclonal bands<br>Disturbet visual evoked potentials	Metilprednisolone pulsterapy	Complete Remission	(Ruggieri M. et al. 1999) [24]
7	F	4y7y	First attack-ataxia, hypoesthesia<br>Second attack (11m)-dysesthesia, chorea<br>Third attack (9m)-optic neuritis<br>Fourth attack (2y)-monoplegia	MRI-mesencephalic and brainstem lesions<br>CSF-peyocitosis, oligoclonal bands<br>Disturbet visual evoked potentials	Metilprednisolone pulsterapy	Complete Remission	(Ruggieri M. et al. 1999) [24]
8	M	4y	First attack-ataxia, headache<br>Second attack (2y)-ataxia, optic neuritis<br>Third attack (6m)-otpic neuritis, headache<br>Fourth attack (8m)- otpic neuritis, headache<br>Fifth attack (10 m)-gait disturbances<br>Sixth attack (3y)-vertigo, optic	MRI-mesencephalicr and brainstem lesions<br>CSF-increased protein level, oligoclonal bands<br>Disturbet visual evoked potentials	Metilprednisolone pulsterapy	Complete Remission	(Ruggieri M. et al. 1999) [24]

			neuritis				
9	M	5y	First attack-ataxia, headache<br>Second attack (1y)-optic neuritis, headache<br>Third attack (10m)-ataxia, dizziness<br>Fourth attack (3y)-diplopia, hemiplegia<br>Fifth attack (10 m)-ataxia, diplopia, optic neuritis<br>Sixth attack (10m)-ataxia, optic neuritis	MRI-periventricular, mesencephalic and brainstem lesions<br>CSF-high IgG, oligoclonal bands<br>Disturbet visual and brainstem auditory evoked potentials	Metilprednisolone pulsterapy	InComplete Remission EDSS=3,5 (poor coordination, gait distrubances)	(Ruggieri M. et al. 1999) [24]
10	F	5y1m	First attack-ataxic gait, diplopia<br>Second attack (4y)-hemiparesis<br>Third attack (6m)-ataxia, diplopia<br>Fourth attack (4m)-palsy, coma<br>Fifth attack (5m)-ataxia, optic neuritis	MRI-periventricular and brainstem lesions<br>CSF-oligoclonal bands<br>Disturbet visual evoked potentials	Metilprednisolone pulsterapy	InComplete Remission EDSS=3 (pyramidal syndrome)	(Ruggieri M. et al. 1999) [24]
11-53	F (25)<br>M (18)	10-23 m (5)<br>2y (15)<br>3y (8)<br>4y (10)<br>5y (7)	Trunck and limb ataxia (30; 61%)<br>Optic neuritis (14; 28%)<br>Seizures (11; 27.,3%)<br>Pyramidal syndrome (9; 18%)<br>Muscle weakness (6; 12%)<br>Headache (4; 8%)<br>Poor gross coordination (3; 6%)	MRI-periventricular (14) subcortical (4) brainstem (8) spinal cord (1) lesions<br>CSF-oligoclonal bands (13), pleocytosis (21) increased protein level (15)	methylprednisolone (5)<br>Prednisolone (13)<br>Other steroids (4)<br>Azathioprine (1)	For 10-23m group:<br>2 died<br>1 with primar y progressive form<br>For the rest:<br>No remission (3)<br>InComplete remission (2)	(Ruggieri M. et al. 1999) [24]

			Coma (2; 4%)				
54	F	2y1m	First attack-ataxia<br>Second attack (8m)-tremor	MRI-periventricular and cerebellar lesions<br>with dissemination in space and time<br>CSF-increased Ac anti-MBP; negative oligoclonal bands and AQP4	methylprednisolone and prednisolone treatment	Prolonged inCompletee remission	(Sivaraman I. et al. 2015) [30]
55	F	3y6m	First attack-ophthalmoplegia<br>Second attack (8m)-ataxic gait	MRI-periventricular, cerebellar and brainstem lesions with evolution in space and time<br>CSF-oligoclonal bands; negative MOGAD, AQP4	methylprednisolone and prednisolone treatment<br>Iv Ig treatment	Completee remission	(Calcii C. et al. 2023) [13]
56	F	2y6m	First attack-fever, ataxic gait<br>Second attack (3m)-paraparesis<br>Third attack (7m)-dysarthria, facial palsy, ophthalmoplegia<br>Fourth attack (1y)- left hemiparesis and facial palsy<br>Fifth attack (1y)- right hemiparesis and facial palsy	MRI-periventricular, peritalamic, cerebellar and brainstem lesions<br>CSF-negative oligoclonal bands, IgG index, MOG, AQP4	methylprednisolone pulse therapy<br>Ig Iv treatment<br>Azathioprine	Complete remission	(Rushdi R. et al. 2024) [25]
57	F	3y	first attack-low fever, right side torticollis, convergent strabismus<br>Second attack (2y9m)-right side divergent strabismuls, dizziness, ataxia	MRI-periventricular and brainstem lesions with dissemination in space and time<br>CSF-oligoclonal bands; ACE negative	methylprednisolone pulse therapy	Complete Remission	(Rai B. et al. 2019) [22]
58	M	4y10m	One attack-gait ataxia, asymmetrical tetraparesis	MRI-periventricular, occipital, brainstem and conus medularis	methylprednisolone pulse therapy	Complete Remission	(Carvalho I. et al. 2023) [11]

				lesions with dissemination in space and time<br>CSF-oligoclonal bands; MOG and AQP4 negative			
59	M	4y	first attack-bilateral optic neuritis<br>Second attack-NOT KNOW	MRI-periventricular lesions with dissemination in space and time<br>CSF-no onligoclonal bands	Metyplrednisolone pulse therapy<br>Interferons	Not know	(Hwang J. et al. 2007) [18]
60	F	4y	first attack-not reported<br>Second, third, fourth attack-NOT KNOW	MRI-periventricular lesions with dissemination in space and time<br>CSF-no onligoclonal bands	Metyplrednisolone pulse therapy<br>Interferons	Not know	(Hwang J. et al. 2007) [18]
61	F	5y	first attack-not reported<br>Second attack-NOT KNOW	MRI-periventricular lesions with dissemination in space and time<br>CSF-no onligoclonal bands	Metyplrednisolone pulse therapy<br>Interferons	Not know	(Hwang J. et al. 2007) [18]
62	F	5y	First attack-ataxia, right hemiparesis,dysphagia, neurologic bladder<br>Second attack (3m)-dyastrha, dysphagia, tetraplegia	MRI-large pericalosal and diencephalic lesions<br>CSF-positive oligcolonal bands and IgG index; negative MOGAD and AQP4	Methylprednisolone pulse therapy<br>Natalizumab	Incomplete remission<br>Complete remission	(Sotgiu S. et al. 2023) [31]
63	M	13m	First attack-right hemiparesis<br>Second attack (5m)-right hemiparesis; flacid paraparesis-spastic transformation<br>Third attack (5 m)-left hemiparesis	CT, MRI-localised periventricular lessions and big flue white matter lessions in the temporo-occipital region<br>CSF-normal cell count, no oligoclonal bands, or IgG index<br>Split V wave on evoked visual potentials	Spontan remission after a month<br>No treatment	Complete remission	(Maeda Y. et al. 1988) [19]
64	M	2y9m	First attack-vomiting, right esotropia, papiledema<br>Second attack (8m)- ataxia, right esotropia	MRI-periventricular lesion with dissemination in space and time<br>CSF-pleiocytosis	Corticosteroids<br>Glatiramer acetat (10 y)	Compelte remission	(Gaccon L. 2006) [14]

			Third-Six attack-in an 8 years time period				
65	F	10m	First attack-meningeal signs<br>Second attack-right hemiparesis<br>Third attack (18m)-meningeal signs<br>Fourth attack (2m)-meningeal signs, left hemiparesis<br>Fifth attack (8m)-amaurosis<br>Six attack (2m)-ptosis, left blindness<br>Last attack (6 years)-paraparesis, seizures, death	CT-white matter low density lesions<br>CSF-pleiocytosis<br>Necropsy-multiple sclerotic cystic lessions	No treatment	Complete remissions of episods<br>Death	(Shaw C-M et al. 1987) [29]
66	M	3y	First attack-altered consciousness, paraparesis<br>Second attack (1y)-hemiparesis, cerebellar symptoms, visual disturbances	MRI-multiple supratentorail and brainstem white matter lesions<br>CSF-no oligoclonal bands or IgG index	Not know	Not know	(Guilhoto L. et al. 1995) [16]
67	M	2y	First attack-hemiparesis, neurologic bladder<br>Second attack (20m)-paraparesis, neurologic bladder<br>Third attack (2y)-hemiparesis, cerebellar symptoms<br>Fourth attack (2y)-monoparesis	MRI-white matter lesions in centrum semioval, corpus callosum<br>CSF-no oligoclonal bands or IgG index	Not know	Not know	(Guilhoto L. et al. 1995) [16]
68	F	2y	First attack-visual disturbances<br>Second attack (5m)-visual	MRI-white matter lesions in right cerebellar peduncle, subcortical and periventricular	Not know	Not know	(Guilhoto L. et al. 1995) [16]

			disturbances<br>Third attack (4m)-visual disturbances<br>Fourth attack (22m)-hemiparesis, cranial nerves involvement<br>Fifth attack (14m)-hemiparesis, cranial nerves involvement<br>Sixth attack (16m)-cerebellar symptoms	CSF-oligoclonal bands and IgG index<br>Disturbet visual evoked potentials			
69	M	2y	First attack-cerebellar symptoms, cranial nerves involvement<br>Second attack (22m)-cerebellar symptoms, cranial nerves involvement<br>Third attack (4m)-visual disturbances<br>Fourth attack (22m)-hemiparesis, cranial nerves involvement<br>Fifth attack (14m)-hemiparesis, cranial nerves involvement<br>Sixth attack (16m)-cerebellar symptoms	CT-white matter lesions adjacent to right frontal horn<br>CSF-pleiocytosis, oligoclonal bands and IgG index<br>Disturbet visual evoked potentials	Not know	Not know	(Guilhoto L. et al. 1995) [16]
70	F	4y	First attack-right hemiparesis<br>Second attack (2y)-vomiting, ataxic gait	MRI-multifocal pattern of white matter lesions<br>CSF-oligoclonal bands, IgG index	Beta-interferon	Complete remission	(Sawant T. et al. 2024) [28]
71	F	3y	First attack- ataxic gait,	CT-small white matter lesions in	Corticotherapy	Complete	(Hauser SL. et

			Second attack (2m)-nystagmus, left Babinsky sign, ataxia<br>Third attack (18m)-fever, nystagmus, left facial palsy, appendicular ataxia, bilateral Babinsky sign, n 6 palsy<br>Fourth attack (4m)-right paraparesis, ataxia, neurologic bladder, focal motor seizure<br>Fifth attack (6y)-ataxia, quadriparesis	the frontal lobes<br>CSF- mild pleocytosis<br>Disturbed visual evoked		remission	al. 1982) [17]
72	F	3y	First attack-hemiparesis<br>Second attack (6y)-cerebellar symptoms, n. 6 palsy<br>Third attack (2m)-right hemiparesis	MRI- characteristic white matter lesions<br>CSF-positive oligoclonal bands<br>Disturbed visual evoked potentials	Not know	Incomplete remission	(Sánchez-Calderón, M. et al. 1998) [27]
73	M	13m	First attack-fever, lethargy, ataxia, paraparesis<br>Second attack (7m)-paraparesis, extrapiramidal syndrome	CT- periventricular and subtentorial hypointense lesions<br>Necropsy- periventricular and cerebellar multiple sclerosis specific lesions	No treatment	Incomplete remission<br>Death	(Cole GF. et al. 1995) [12]
74	M	3y3m	First attack-right hemiparesis<br>Second attack (4y)-right monoparesis of the inferior limb<br>Third attack (4y3m)-right monoparesis of the inferior limb	MRI-areas of T2 hyposignal in the posterior part of both semioval centers and in right dentate nucleus<br>Normal visual evoked potentials	ACTH treatment	Compelte remission	(Rodríguez Núñez A.; et al. 1992) [23]
75-101	F (16)	2-5y	Optic neuritis (6; 22%)<br>Seizures (11; 27.,3%)	MRI-periventricular (18) subcortical (16) brainstem (12),	Not know	Incomplete remission (5)	(Mikaeloff, Y. et al. 2006)

	M (11)		Pyramidal syndrome (20; 74%)<br>Brainstem dysfunction (8; 30%)<br>Transverse myelitis (3; 11%)	thalamus (12), spinal cord (6) lesions<br>CSF-oligoclonal bands (4), pleocytosis (16) increased protein level (10)			[20]
